# Standard-Deviation-Based Adaptive Median Filter for Elimination of Batwing Effects in Step Microstructure Measurement Using Digital Holography

**DOI:** 10.3390/s24185928

**Published:** 2024-09-12

**Authors:** Jiasi Wei, Junjie Wu, Chen Wang

**Affiliations:** 1 Key Laboratory of Bioanalysis and Metrology for State Market Regulation, Shanghai Institute of Measurement and Testing Technology, Shanghai 201203, China; 2 Department of Precision Mechanical Engineering, Shanghai University, Shanghai 200444, China

**Keywords:** digital holography, batwing effect, step microstructure measurement, optical metrology

## Abstract

Digital holography has transformative potential for the measurement of stacked-chip microstructures due to its non-invasive, single-shot, full-field characteristics. However, significant light scattering and diffraction at steep edges in step microstructures cause the batwing effect, leading to measurement errors. Herein, we propose a standard-deviation-based adaptive median filter to eliminate batwing effects in step microstructure measurement using digital holography. The standard deviation determines the positions of the steps and the range of the batwing effect. During filtering, the filter window size varies: it adjusts according to the center’s position within the batwing effect range and reduces outside this range to prevent distortion in other regions. Filtering weights are set to maintain information integrity while using larger filter windows. Experiments on the Standard Resolution Target USAF 1951 and the standard step height target show that our method successfully eliminates batwings while preserving the integrity of the remaining profile.

## 1. Introduction

In recent years, there has been a significant trend towards the miniaturization of components within the fields of aerospace, integrated circuits, and optical manipulation [1,2,3]. Consequently, high-resolution surface topography measurement has become increasingly crucial in ensuring product quality control and monitoring manufacturing processes [4,5]. Digital holography has emerged as a powerful tool in the precise measurement of microstructures, particularly in applications involving stacked-chip configurations [6]. Its non-invasive nature, coupled with single-shot and full-field capabilities, makes it an attractive method for the capture of detailed three-dimensional information [7,8]. This advanced optical technique enables the reconstruction of the amplitude and phase of an object’s wavefront, providing high-resolution imaging that is crucial for various scientific and industrial applications [9,10]. However, one significant issue encountered during the measurement of step microstructures is the batwing effect, a non-linear phenomenon that arises due to light scattering and diffraction at the steep edges of these structures [11,12].. This scattered and diffracted light interferes with itself and the reference beam, leading to inaccuracies in the recorded holographic data [13]. The batwing effect is particularly problematic as it introduces non-linear artifacts that can obscure the true topography of the microstructure.

Recent studies have explored various approaches to improve the accuracy and reliability of optical measurement in the presence of such artifacts. Xie et al. developed a hybrid filtering approach combining spatial and frequency domain filters to mitigate the impact of batwing effects in interferometric measurements, resulting in more accurate topographical data [14]. Lehmann et al. introduced an innovative phase compensation method to correct for the phase errors in vertical scanning white-light interferometry, which can effectively reduce batwing effects and improve surface topography measurements [15]. Sun et al. proposed adaptive algorithms and advanced filtering techniques that have shown promise in addressing measurement errors caused by diffraction and scattering [16]. Moreover, research into phase retrieval methods and image reconstruction has provided valuable insights into reducing noise and enhancing the image quality [11]. Li et al. proposed an algorithm based on Haar wavelet transforms to detect and eliminate the batwing effect in white-light scanning interferometry, effectively reducing systematic errors without significantly increasing the time complexity [12]. Huang et al. provided a novel approach that combines a free field of view scheme with infrared holographic detection, which enables the acquisition of phase attributes [17]. These advancements highlight the ongoing efforts to eliminate batwing effects for complex microstructures measured using optical metrology methods. However, the current filtering techniques often fail to address these artifacts effectively without compromising the integrity of the surrounding data. Therefore, there is a critical need for advanced filtering methods that can selectively mitigate the batwing effect while preserving the fidelity of the overall measurement.

Based on our previous work on digital holographic measurement [6] and step height characterization [18,19], we propose a novel approach using a standard-deviation-based adaptive median filter (SAMF) to eliminate batwing effects in step microstructure measurement via digital holography. Our method leverages the standard deviation to accurately determine the positions and range of the batwing effect, allowing for the adaptive adjustment of the filtering window size. When the filter matrix center lies within the batwing effect range, the window size is dynamically altered based on its position. Outside this range, the window size is reduced to prevent the distortion of other areas of the object. Additionally, we introduce filtering weights to ensure that the information remains undistorted when using larger filter windows within the batwing effect range.

## 2. SAMF for Batwing Elimination

### 2.1. Digital Holography for Microstructure Measurement

**Figure 1:**
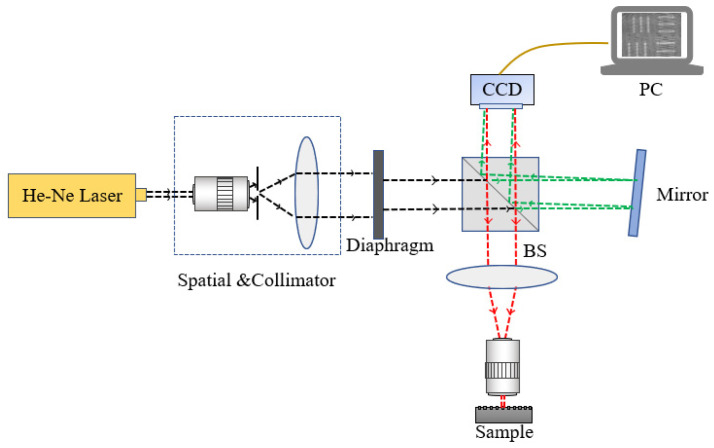
Digital holography system for measurement of microstructures.

The microstructure measurement system is illustrated in Figure 1. It is a digital off-axis Michelson system, using a He-Ne laser with a wavelength of 632.8 nm as the illumination source. After the beam’s expansion and collimation, it is directed onto the measurand. The digital hologram is recorded via a CCD camera. The specific information of the optical elements is introduced in the experimental part in Section 3. The phase is retrieved according to the Fresnel diffraction reconstruction principle in digital holography, which is described in detail in our previous work [6].

### 2.2. Sources of Batwing

In interferometric measurements, the most direct factor contributing to the error of the batwing effect is the diffraction of light. The top (or bottom) diffraction signals at the edges of the step are superimposed onto the bottom (or top) diffraction signals, affecting the recording of the optical interference signal. In holography, the recording of holographic images involves interference between the diffracted light from the object and the reference light. This results in the occurrence of overlapping information at the edges of the step in the holographic image, preventing the accurate demodulation of the morphology and leading to the appearance of batwings in the reconstructed image.

For the holographic measurement system that we have developed, the topography associated with the edges of the step (square wave) spans a considerable frequency spectrum. The superimposition of the top (or bottom) diffraction signals at the edges of the step onto the corresponding bottom (or top) diffraction signals significantly impacts the recording of optical interference signals. Consequently, the holographic image exhibits overlapping information at the edges of the step, impeding the accurate demodulation of the morphology and leading to batwing artifacts in the holographic reconstruction. Moreover, in off-axis digital holography, the requisite selection of the positive first-order spectrum necessitates the careful consideration of the filtering window, as an inappropriate choice may truncate the frequency components of the square wave, engendering “ringing artifacts” and thereby contributing to the observed batwing effect.

In instances where the step is non-transparent to the probing light, the attainment of a certain step height results in the occlusion of the detection light, thereby diminishing the numerical aperture (NA) of the detection system. This reduction in NA, in turn, diminishes the frequency response of the detection system, yielding a pseudo-resolution effect concerning the morphology proximal to the edges. Achieving the precise resolution of the edges of the step necessitates an ample distribution of sampling points in the vicinity of the step corners. However, owing to constraints imposed by the NA of the objective and the discrete nature of CCD detectors, an inevitable lack of sampling points is encountered in the vicinity of the step corners.

Furthermore, secondary factors influencing the measured results of the batwing effect encompass considerations related to the light source characteristics, objective specifications, ambient light interference, external vibrational disturbances, fluctuations in the intensity of the light source, etc.

In measuring the topography of a step, if the intensity distribution of light within the interference objective is uniform, then $\theta_{0}=\arcsin(NA)$. Assuming that the height of the topography is represented by h(x,y), the intensity value of the interference light is given by

(1) $$I(x,y)=4\pi k^{2}{\left|U\right|}^{2}\int_{0}^{\theta_{0}}\left\{A^{2}+B^{2}+2AB\cos\left[2kh(x,y)\cos\theta +\varphi \right]\right\}\sin\theta \cos\theta d\theta$$

where *U* represents the spatial Fourier transform of the optical field distribution, *A* and *B* are the light waves from the reference and object, and *φ* is the phase change caused by the reflection of the object light wave. $A^{2}+B^{2}$ is the direct current component of the interference light intensity, namely the background light intensity. $2AB\cos\left[2kh(x,y)\cos\theta +\varphi \right]$, representing the change in the intensity and peak position of the interference light, is the alternating current component of the interference light. Therefore, in our study, we only consider the alternating current component:

(2) $$I(x,y)=8\pi k^{2}{\left|U\right|}^{2}\int_{0}^{\theta_{0}}\left\{AB\cos\left[2kh(x,y)\cos\theta +\varphi \right]\right\}\sin\theta \cos\theta d\theta$$

During interferometric recording, the most direct factor leading to the error of the batwing effect is the diffraction of light. At the steep edges of the step, incident light diffracts to the bottom and top of the step and reflects back. The information from the reflected light at the bottom and top combines and returns along the original path to the interference objective. This results in at least three components influencing the interference signal on the imaging plane at the edge of the step: the reflected light from the object under measurement, the reference light, and the diffracted light from the vicinity of the top (or bottom) plane of the step. Therefore, considering the impact of diffraction, we obtain the two-dimensional point spread function of the non-coherent imaging Airy disk:

(3) $$psf(r)={\left[\frac{2J_{1}(u(r))}{u(r)}\right]}^{2}$$

where $r=\sqrt{\left(x^{2}+y^{2}\right)}$, *J*_1_ refers to the first type of Bessel function, u(r)=2π⋅NA⋅r/λ.

The light intensity information at each sampling point on the sample is convolved with the point spread function of the interference objective, thereby obtaining an interference intensity image that takes into account diffraction by Equation (4), in which ⊗ denotes the convolution operator:

(4) $$I_{CCD}(x,y)=I(x,y)⊗psf(x,y)$$

It is worth noting that, in the equation, $I_{CCD}(x,y)$ takes into account diffraction effects, representing the interference intensity signal observed on the Charge-coupled Device (CCD) in practice. According to the principles of diffraction, it can be observed that, in the actual measurement of interferometric fringe recording, the light intensity information at the measurement point includes not only the reference and object light waves in the interference optical path but also the superposition of diffracted light around the measurement point.

### 2.3. Principle of SAMF

As the batwing effect is a non-linear phenomenon, median filtering is employed for its suppression. For the profile, the median value within an array is taken as the output result, expressed by the following equation:

(5) $$Y[n]=Median\left\{X[n-l],\ldots ,X[n],\ldots ,X[n+l]\right\},n\in N,l=\frac{k-1}{2}$$

where “*Median*” denotes the calculation of the median value from the data. *X* represents the raw measurement data, *Y* signifies the filtered data, *n* denotes the sampling position, *k* represents the width of the filtering window, and $l=\frac{k-1}{2}$ denotes half of the filtering window width.

For areal topography, the median filtration is

(6) $$Y(i,j)=Median\left(\begin{array}{ccc} X(i-l,j-l) & \ldots & X(i-l,j+l)\\ \vdots & X(i,j) & \vdots \\ X(i+l,j-l) & \cdots & X(i+l,j+l) \end{array}\right),\mathrm{and}\;i,j\in N,l=\frac{k-1}{2}$$

where X(i,j) represents the center of the filtering matrix, and the filtered data Y(i,j) will replace the original data at the corresponding position; *k* denotes the width of the filtering window, and $l=\frac{k-1}{2}$ represents half of the window width.

The batwing range is determined by the standard deviation, which is generated from the raw measurement data. Initially, along a certain dimension, assuming that the batwing range at a particular step edge is denoted by X[m:m+width,q], when the center of the filtering matrix, X[p,q], is not within the batwing range, the width *k* of the filtering window in the above expression takes its default value, $k_{default}=3$. When the center lies within the batwing range, the half-width of the filtering window, $k=k_{default}+\min\left\{\left|p-m\right|,\left|m+width-p\right|\right\}$, is employed. Consequently, when positioned within the batwing range, the size of the filtering window will vary with changes in the position of the filtering matrix center.

Furthermore, a discriminant threshold is established, typically set as the mean of the raw data within the batwing range, to distinguish whether the filtering matrix data belong to a high or low step and assigning weights. If the data within the filtering window exceed (fall below) this threshold, the corresponding data will be set to zero during filtering, preventing the data from being influenced by the relatively large batwing data within the range.

The procedure of our proposed Algorithm 1 can be outlined as follows.
**Algorithm 1** Suppression of the batwing effect in microstructure measurement(i)Generate the standard deviation image from the original measurement data;(ii)Select an appropriate batwing range based on the standard deviation image;(iii)Automatically determine the window size of the filtration: when the center data are not within the batwing range, use the default size, 3; when the center data are within the batwing range, the size will change with the alteration of the center data position;(iv)Set a threshold; when the center data are greater than (less than) this threshold, set the matrix values smaller than (larger than) this threshold to zero;(v)Sort and take the median of the non-zero data in the filtering matrix;(vi)Iterate through all data points to obtain the filtered data**end**

In summary, the standard deviation is utilized to determine the position of the step in microstructure surfaces and the range of the batwing effect. During the filtering process, the size of the filtering window is variable. Within the batwing effect range, the window size changes based on the alteration of the filtering matrix center position, ensuring that the window size decreases outside the batwing effect range. This adjustment prevents the distortion of information in other areas of the surface. Additionally, filtering weights are set to minimize distortion when applying a large-sized filtering window to filter the batwing effect.

## 3. Experimental Studies and Analysis

**Figure 2:**
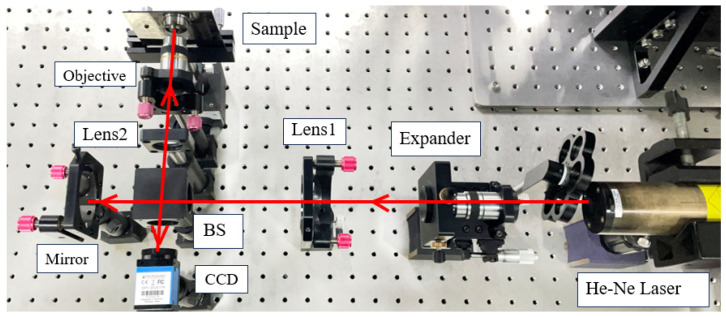
The optical configuration of the developed measurement system.

A digital holographic system based on the principles illustrated in Figure 1 was developed. Its corresponding optical configuration is detailed in Figure 2. The expander and Lens 1 are used for beam expansion and collimation, and Lens 2 works as a tube lens to achieve clearer holograms in CCD. Two distinct experiments were conducted, involving the measurement of the Standard Resolution Target USAF 1951, provided by the company Thorlabs, and the standard step height provided by the National Institute of Metrology of China (NIM).

The measurements were conducted in a controlled laboratory environment with a maintained temperature of (20 ± 0.5) °C and relative humidity of (50 ± 5)% RH, utilizing an air suspension optical isolation platform to ensure effective vibration isolation. The He-Ne laser utilized had a wavelength of 632.8 nm, and the CCD camera, a product of The Imaging Source, was of the type DMK 33GX273, possessing 1440 × 1280 pixels with a pixel size of 3.45 μm. The imaging size of the CCD camera was 4968 × 4416 μm^2^

### 3.1. Experiment 1: Measuring the Standard Resolution Target USAF 1951

**Figure 3:**
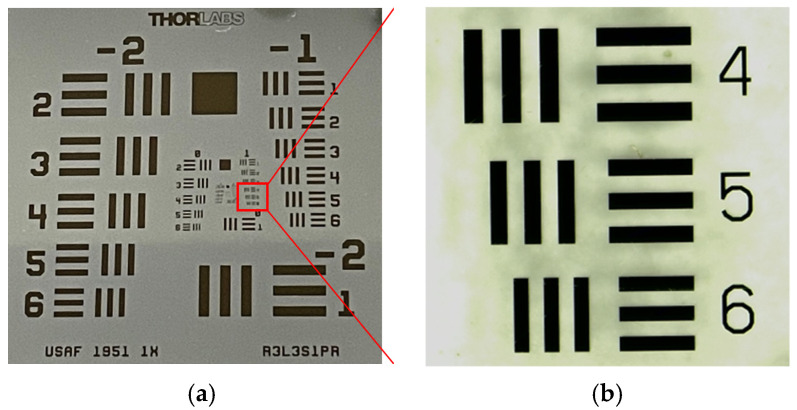
Photograph of the Standard Resolution Target USAF 1951: (**a**) the whole view; (**b**) the measurement area.

The Standard Resolution Target USAF 1951 is shown in Figure 3a, with the measurement area identified as the first group and fifth element, indicated in the red rectangle. A magnified view of the measurement area is shown in Figure 3b, which was obtained with a traditional optical microscope.

**Figure 4:**
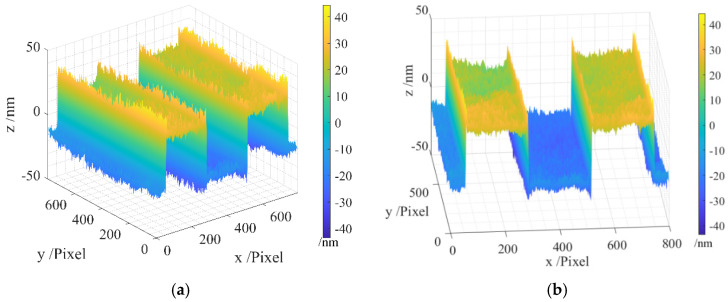
Areal topography of the measurement area of the resolution target before filtration: (**a**) 45-degree view; (**b**) front view.

The areal topography of the measured region of the resolution target is presented in Figure 4, wherein Figure 4a gives a 45-degree view, and Figure 4b provides a front view. It can be observed that the original measurement data contain a batwing, particularly noticeable at the four edges of the step, introducing considerable noise.

**Figure 5:**
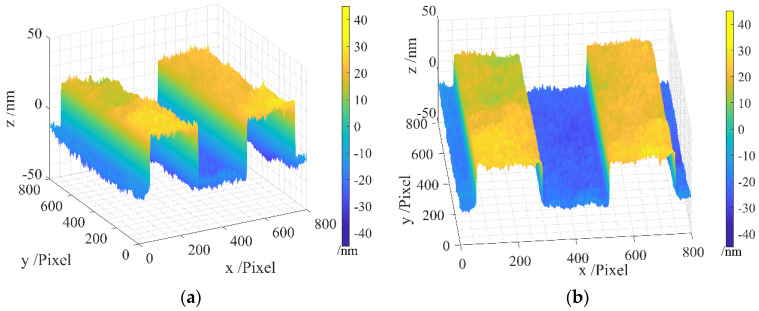
Areal topography after filtration: (**a**) 45-degree view; (**b**) front view.

After applying our method for batwing elimination, the batwing is successfully removed, as shown in Figure 5a,b, presenting the same result from both the 45-degree and front views.

**Figure 6:**
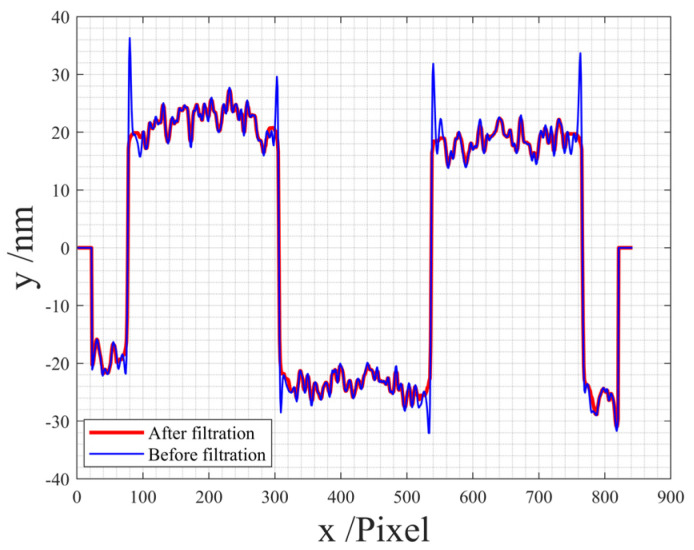
Comparison of the 2D profiles before (in red) and after (in blue) filtration.

Additionally, a profile is drawn for comparison, as illustrated in Figure 6. The blue profile represents the original profile before applying our batwing elimination method, while the red profile reflects the outcome after the application of our proposed method.

A comparative analysis between Figure 4 and Figure 5 substantiates that our proposed method accurately eliminates the batwing. The examination of the original profile and the profile after applying our batwing elimination method in Figure 6 reveals the successful elimination of the batwing at the edges, while the remainder of the profile remains unchanged. These results affirm the feasibility and efficacy of our proposed method.

### 3.2. Experiment 2: Measurement of the NIM Standard Artifact

**Figure 7:**
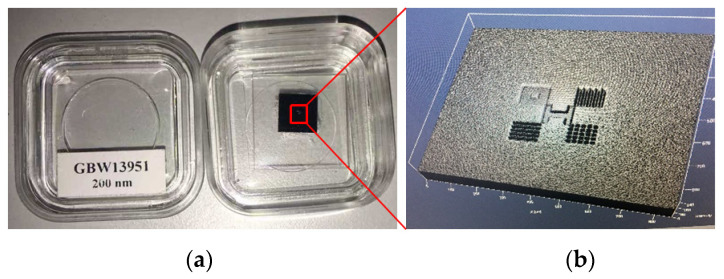
NIM-provided standard: (**a**) photograph; (**b**) surface at 50X magnification captured by confocal microscopy, Zeiss LSM 710.

The step height standard artifact is shown in Figure 7. The standard comprises five patterns, each with a nominal step height of 200 nm. Calibration conducted by the NIM yielded a calibrated value of 209.7 nm, with uncertainty *U*_95_ of 2.3 nm.

**Figure 8:**
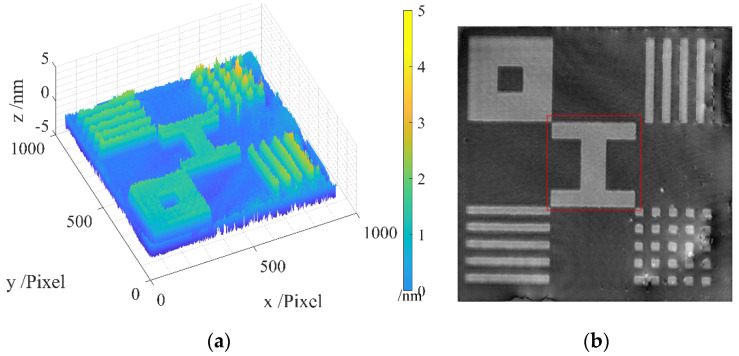
Raw measurement results: (**a**) areal topography; (**b**) intensity image with the designated area for batwing elimination highlighted in the red square.

The raw measurement results of the standard are presented in Figure 8a, encompassing five patterns. The middle pattern, highlighted within the red square in Figure 8b, is chosen for batwing elimination using our proposed method.

**Figure 9:**
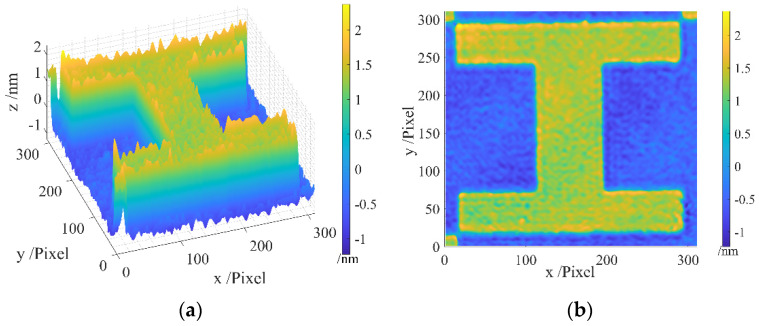
Areal topography of the measurement area of the standard artifact before filtration: (**a**) 45-degree view; (**b**) top-down view.

The designated area for batwing elimination is illustrated in Figure 9, presenting both a 45-degree and a top-down view. In the 45-degree view, noticeable batwings are observed on the step edges. In the top-down view, the edges exhibit distinct colors, as indicated in the color bar, signaling the presence of batwings.

**Figure 10:**
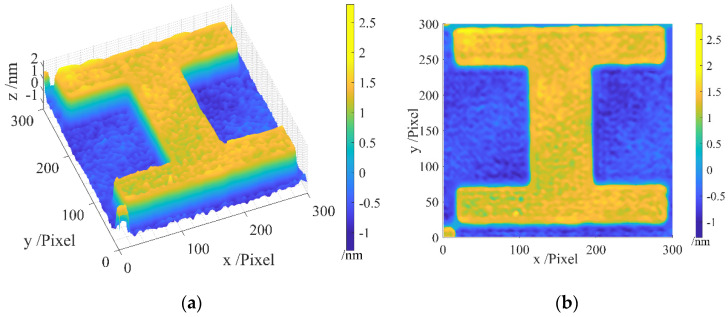
Areal topography of the measurement area of the standard artifact after filtration: (**a**) 45-degree view; (**b**) front view.

After applying our batwing elimination method, the results are as given in Figure 10, featuring a 45-degree view and a top-down view. In Figure 10a, no batwings are evident on the edges. Figure 10b reveals the more uniform color distribution across the entire pattern area, with no batwings discernible on the edges.

**Figure 11:**
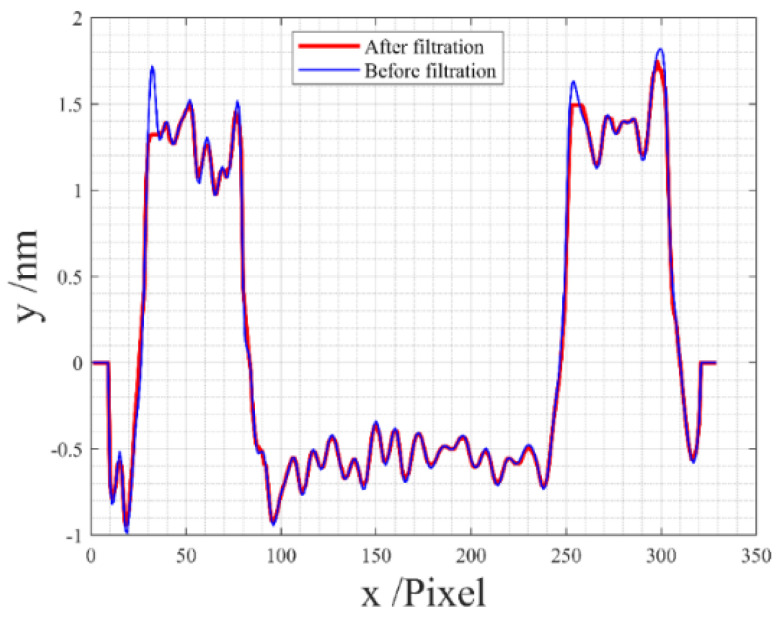
Comparison of the 2D profiles before (in red) and after (in blue) filtration in Experiment 2.

Additionally, a profile is drawn for comparative analysis, as shown in Figure 11, where the blue profile represents the original, and the red profile signifies the result after applying our proposed method. It is evident that the batwings on the edges are eliminated, while the remainder of the profile remains consistent, as indicated by the alignment of the red and blue profiles.

A comparison between Figure 9 and Figure 10 demonstrates the effectiveness of our method in removing batwings. The results in Figure 11 further affirm that our method successfully eliminates batwings while preserving the integrity of the remaining profile. These results establish the feasibility and effectiveness of our proposed method.

## 4. Conclusions

This study introduced a standard-deviation-based adaptive median filter (SAMF) to eliminate batwing effects in step microstructure measurements using digital holography. It uses the standard deviation to identify step positions and the batwing effect range. The adaptive filter adjusts its window size dynamically. It changes within the batwing effect range based on the center’s position and reduces outside this range to prevent distortion in other regions. Filtering weights ensure information integrity even with larger windows. Experiments on the Standard Resolution Target USAF 1951 and the step height standard artifact confirm that our method effectively eliminates batwing effects while preserving the profile’s integrity. The results demonstrate that our filter improves the accuracy in digital holography measurement, enhancing its application in precision metrology and high-resolution surface characterization.

## Data Availability

Data are contained within the article.
